# Assessment of myocardial fibrosis in ischemic and non-ischemic cardiomyopathies using cardiac magnetic resoance non-contrast T1ρ mapping

**DOI:** 10.3389/fcvm.2026.1797516

**Published:** 2026-04-13

**Authors:** Yufan Gao, Anhong Yu, Shuo Liang, Yanhe Ma, Boxin Li, Hong Zhang, Zhigang Guo

**Affiliations:** 1 Academy of Medical Engineering and Translational Medicine, Tianjin University, Tianjin, China; 2 Department of Radiology, Chest Hospital, Tianjin University, Tianjin, China; 3 Department of Cardiac Surgery, Chest Hospital, Tianjin University, Tianjin, China; 4 Tianjin Key Laboratory of Cardiovascular Emergency and Critical Care, Tianjin Municipal Science and Technology Bureau, Tianjin, China

**Keywords:** cardiac magnetic resonance, cardiomyopathy, late gadolinium enhancement, myocardial fibrosis, T1-rho

## Abstract

**Aim:**

To evaluate the performance of T1ρ mapping for myocardial fibrosis detection across distinct cardiomyopathy entities of ischemic and non-ischemic origin against native T1 mapping.

**Methods and results:**

A total of 14 healthy controls and 39 patients [15 with ischemic cardiomyopathy [ICM], 12 with hypertrophic cardiomyopathy [HCM], and 12 with dilated cardiomyopathy [DCM]] underwent cardiac magnetic resonance (CMR), including T1ρ mapping, native T1 mapping, late gadolinium enhancement (LGE), and extracellular volume (ECV) mapping. Segments were visually classified as segments with or without LGE. T1ρ values were significantly higher in patients with ICM, HCM, and DCM than controls (all *P* < 0.05). T1ρ showed favorable diagnostic performance compared with native T1 in distinguishing ICM and HCM patients from controls [area under the curve (AUC): 0.959 vs. 0.739 for ICM; AUC: 0.878 vs. 0.763 for HCM], while both parameters exhibited comparable performance in DCM (AUC: 0.814 vs. 0.814). Notably, elevated T1ρ values were observed in segments with or without LGE. T1ρ mapping demonstrated good performance in distinguishing segments with LGE from those without LGE in ICM and HCM (AUC: 0.820 and 0.726) and exhibited a significant correlation with ECV across all disease types (ICM: *r* = 0.598; HCM: *r* = 0.577; DCM: *r* = 0.648; all *P* < 0.05).

**Conclusion:**

This exploratory study demonstrates the potential of T1ρ mapping as a non-contrast CMR technique for detecting myocardial fibrosis in ischemic and non-ischemic cardiomyopathies. T1ρ showed favorable diagnostic performance compared with native T1 in ICM and HCM with comparable performance in DCM. These findings warrant validation in larger cohorts with diverse cardiac conditions to establish the clinical utility of T1ρ imaging.

## Introduction

Myocardial remodeling is a common pathological process observed across various cardiac diseases. A hallmark of myocardial remodeling is the development of myocardial fibrosis, which is associated with adverse outcomes (1, 2). Late gadolinium enhancement (LGE) is a widely used technique for detecting myocardial fibrosis (3, 4), with both visual assessment and various quantitative approaches being used for its interpretation. However, LGE requires the administration of gadolinium-based contrast agents, which can pose risks to patients with renal impairment and are additionally deposited in brain (5, 6). Moreover, LGE has limited sensitivity in detecting diffuse fibrosis, as it may be impossible to define an area of clearly unaffected myocardium as a “nulled” reference in diffuse fibrotic processes. Cardiac magnetic resonance (CMR) quantitative parametric mapping has emerged as a promising non-invasive tool for myocardial tissue characterization, the diagnosis and prognosis of various cardiac diseases, and therapeutic efficacy monitoring (7, 8). However, elevations in native T1 are influenced by a multitude of factors and are not specific to fibrosis (9). Additionally, T1 mapping-derived extracellular volume (ECV) fraction, as a reference marker for assessing myocardial diffuse fibrosis with higher repeatability and comparability than native T1 (10), requires calculation based on the T1 mapping value before and after contrast agent injection.

T1ρ mapping has been gaining attention as a novel endogenous biomarker for the quantitative evaluation of myocardial injuries (8). T1ρ-relaxation describes the longitudinal relaxation in a rotating frame, under a spin lock (SL) radiofrequency pulse. The T1ρ relaxation time is sensitive to low-frequency interactions between bulk water and macromolecules (8). In fibrotic myocardium, collagen deposition alters the local macromolecular environment and water-proton interactions, leading to changes in these low-frequency exchange processes and consequently altering T1ρ relaxation times. Based on this mechanistic link, T1ρ mapping has been proposed as a potential non-contrast technique for detecting myocardial fibrosis in ischemic and non-ischemic heart diseases (11–13); however, its clinical implementation remains in its infancy, with limited *in vivo* investigations across diverse cardiac conditions. Segmental analysis offers detailed insights into local tissue characteristics, enabling the detection of subtle regional difference.

Therefore, this study aims to evaluate the performance of T1ρ mapping for myocardial fibrosis detection across distinct cardiomyopathy entities of ischemic and non-ischemic origin against native T1 mapping.

## Methods

### Study population

From May 2024 to October 2024, 39 patients undergoing CMR in our institution were prospectively included. The inclusion criteria were as follows: (a) ischemic cardiomyopathy (ICM) was defined by the presence of reduced left ventricular (LV) ejection fraction (EF) (<50%), a typical subendocardial or transmural scar on LGE imaging, ≥70% stenosis in ≥1 epicardial coronary vessels on angiography, or history of myocardial infarction or coronary revascularization. (b) hypertrophic cardiomyopathy (HCM) was diagnosed by the presence of a non-dilated and hypertrophied LV at CMR (≥15 mm in adults or ≥13 mm in family member of HCM patients) in the absence of another disease that could account for the hypertrophy (14). (c) And, dilated cardiomyopathy (DCM) was diagnosed based on the presence of impaired systolic function (LVEF < 50%) and LV dilation [increased LV end-diastolic volume indexed to body surface area compared with published gender-specific reference values (15)], in the absence of coronary artery disease or abnormal loading conditions (16). The exclusion criteria were age <18 or poor imaging quality. In addition, the study recruited individuals without cardiovascular risk factors, diseases, or medications as controls. This study was approved by the Medical Ethics Committee of Chest Hospital, Tianjin University, and written informed consent was obtained from all participants.

### CMR acquisition

All participants underwent CMR on a 3.0 T MRI system (Philips Health Care, Ingenia, Netherlands). Cardiac short-axis, 2-chamber, 3-chamber, and 4-chamber view images were acquired using a standard breath-hold steady-state free precession cine sequence prior to the administration of gadolinium contrast agent with the following parameters: slice thickness, 8 mm; gap, 2 mm; repetition time (TR), 2.8 ms; echo time (TE), 1.4 ms; field of view, 300 × 300 mm^2^; 25 phases per cardiac cycle. LGE images were acquired using phase-sensitive inversion recovery sequence 10–15 min after administration of gadolinium-based contrast agent in the two-chamber, four-chamber and a series of LV short-axis views. Native and 15 min post-contrast T1 mapping were acquired in basal, mid, and apical LV short-axis slices using a modified Look-Locker inversion recovery (MOLLI) sequence. Imaging parameters were: slice thickness, 8 mm; TR, 2.1 ms; TE, 1.0 ms; field of view, 300 × 300 mm^2^; acquisition scheme, 5s(3s)3s for native T1 mapping and 4s(1s)3s(1s)2s for post contrast T1-mapping.

T1ρ mapping was performed pre-contrast at the same basal, middle, and apical levels of the LV short axis as the T1 mapping slices using a T1ρ-prepared balanced steady-state free precession (bSSFP) sequence. The T1ρ pulse cluster consisted of 90_x_-SL_y_-180_y_-SL_−y_-90_−x_, with dual spin-locks with opposite phases and a refocusing pulse between the spin-locking halves to compensate for B1 variations and B0 errors (17). Four T1ρ-weighted images with different time of spin locking [TSL = (0, 13, 27, 40) ms] were acquired sequentially in mid-diastole during 10 heartbeats (with a repetition time of three heartbeats to allow for enough magnetization recovery) in a single breath-hold, with electrocardiogram triggering. The scanning parameters are as follows: spin-lock frequency, 350 Hz; slice thickness, 8 mm; flip angle, 35°; TR, 1.90 ms; TE, 0.60 ms; matrix size, 135 × 135; field of view, 270 × 270 mm^2^; bandwidth, 1,875.8 Hz/pixel; sensitivity encoding (SENSE) factor, 2. All the CMR parameters are summarized in Table 1.

**Table 1:** Parameters of cardiac magnetic resonance images.

Parameter	Cine	T1 mapping	LGE	T1ρ mapping
Slice thickness (mm)	8	8	8	8
Field of view (mm^2^)	300 × 300	300 × 300	300 × 300	270 × 270
Matrix size	256 × 256	150 × 150	187 × 158	135 × 135
Flip angle (°)	45	20	15	35
TR (ms)/TE (ms)	2.8/1.4	2.1/1	5.2/2.6	1.9/0.6
SENSE	1.5	1.5	1.5	2
Bandwidth (Hz/pixel)	1,775.6	1,082.3	306.4	1,875.8
Spin-lock frequency (Hz)	–	–	–	350
TSL	–	–	–	[0, 13, 27, 40]

LGE, late gadolinium enhancement; TR, repetition time; TE, echo time; SENSE, sensitivity encoding; TSL, time of spin locking.

### CMR analysis

Imaging analysis was performed using cvi42 software (version 5.14.0, Circle Cardiovascular Imaging Inc., Calgary, AB, Canada). LV function were assessed by automatically tracing the LV endocardial and epicardial borders on short-axis cine images at the end-systolic and end-diastolic phases, with manual correction as needed. LV function parameters were then recorded, including LVEF, stroke volume, cardiac output, LV end-diastolic volume (LVEDV), LV end-systolic volume (LVESV), and LV mass (LVM), all indexed to body surface area (BSA) using the Mosteller formula (18). LVM was determined at end-diastole, excluding papillary muscles.

The LV endocardial and epicardial borders were semi-automatically delineated on T1 and T1ρ maps for three short-axis slices, avoiding the potential contamination from blood pool and extra-myocardial structures. ECV was calculated using the formula: ECV = (ΔR1myocardium/ΔR1blood) × (1 − hematocrit), where R1 = 1/T1. Measurements were reported on the 16-segment American Heart Association (AHA) model (18) and averaged to provide a global value. Segments were visually classified as segments with LGE (LGE+), or segments without LGE (LGE–). The raw T1ρ-weighted images and corresponding T1ρ maps were examined to assess for artefacts caused by incorrect motion correction and poor shimming. All control subjects were excluded from the ECV and LGE analyses since they did not receive contrast agent due to potential risks.

Intra- and inter-observer variability for parameters were assessed using the intraclass correlation coefficient (ICC). The same observer analyzed the same 20 randomly selected individuals twice at 2-week intervals to determine intra-observer variability. Inter-observer variability was assessed by two independent, blinded observers analyzing the same 20 individuals.

### Statistical analysis

Continuous variables were presented as mean ± standard deviation or median (interquartile range). Categorical variables were expressed as percentages and compared using the Chi-square test or Fisher's exact test. Normality testing was performed using the Shapiro–Wilk test. Student's *t*-test, one-way analysis of variance (ANOVA), and Kruskal–Wallis test (with appropriate *post hoc* analysis adjusted with the Bonferroni correction) performed as appropriate for comparisons between groups for continuous variables. The correlation between T1ρ and other tissue characteristics was assessed using Pearson's or Spearman's correlation coefficients as appropriate. Generalized linear mixed effect models (GLMMs) with random intercepts for each person and each segment were employed to account for the fact that different segments from the same patient or the same segment location from different patients may exhibit correlated behavior. The models assessed the association between tissue characteristics and LGE. Additionally, receiver operating characteristic (ROC) analysis was conducted to evaluate the diagnostic accuracy of tissue characteristics in differentiating patients from healthy controls and differentiating LGE+ segments from LGE− segments. For comparisons of AUCs between T1ρ and native T1, the DeLong test was used. A *P*-value < 0.05 was considered statistically significant.

## Results

### Patient characteristics

A total of 14 controls and 39 patients were included in the study. The patient group comprised 15 patients with ICM, 12 with HCM, and 12 with DCM. The baseline characteristics of the study population are shown in Table 2. There was no significant difference in BSA and heart rate among the groups.

**Table 2:** Baseline characteristics.

Variable	Controls (*n* = 14)	ICM (*n* = 15)	HCM (*n* = 12)	DCM (*n* = 12)	*P* value
Age (years)	52.14 ± 7.83	61.53 ± 13.39	55.92 ± 20.16	52.33 ± 11.91	0.242
Female, *n* (%)	10 (71.4)	3 (20.0)	8 (66.7)	5 (41.7)	0.022
BSA (m^2^)	1.71 ± 0.13	1.79 ± 0.17	1.80 ± 0.25	1.86 ± 0.19	0.230
Heart rate (bpm)	74.64 ± 10.49	68.80 ± 12.19	65.83 ± 8.62	71.42 ± 8.75	0.170
Hematocrit (%)	-	41.17 ± 5.29	42.08 ± 3.56	44.29 ± 6.21	0.296
Comorbidities					
Hypertension, *n* (%)	0 (0.0)	9 (60.0)	8 (66.7)	3 (25.0)	<0.001
Diabetes mellitus, *n* (%)	0 (0.0)	3 (20.0)	1 (8.3)	2 (16.7)	0.370
Smoking, *n* (%)	2 (14.3)	11 (73.3)	3 (25.0)	2 (16.7)	0.002
Hypercholesterolemia, *n* (%)	0 (0.0)	3 (20.0)	1 (8.3)	3 (25.0)	0.189

Data are expressed as mean ± standard deviation or *n* (%). ICM, ischemic cardiomyopathy; HCM, hypertrophic cardiomyopathy; DCM, dilated cardiomyopathy; BSA, body surface area.

### CMR measurements

CMR measurements for all subjects are shown in Table 3. Patients with ICM, HCM and DCM had higher LVM index compared with controls (all *P* < 0.01), with no significant differences between any of the three patient groups (all *P* > 0.05). The ICM group and DCM group showed lower LVEF and greater LVEDV index and LVESV index than the control group (all *P* < 0.05). The HCM group showed significantly greater stroke volume index than other three groups (all *P* < 0.01), with no significant differences between any of the other three groups (all *P* > 0.05).

**Table 3:** CMR findings of healthy controls and patients.

Variable	Control (*n* = 14)	ICM (*n* = 15)	HCM (*n* = 12)	DCM (*n* = 12)	*P* value
Left ventricular function					
LVEF (%)	64.90 ± 6.20	37.11 ± 10.89^a^	67.44 ± 9.34^b^	29.94 ± 9.30^a^^,^^c^	<0.001
Stroke volume index (mL/m^2^)	36.55 ± 4.88	31.44 ± 10.24	49.09 ± 7.92^a^^,^^b^	36.15 ± 8.21^c^	<0.001
Cardiac index (L/min/m^2^)	2.73 ± 0.55	2.11 ± 0.60	3.25 ± 0.77^b^	2.59 ± 0.72	0.001
LVEDVI (mL/m^2^)	56.23 ± 3.73	92.04 ± 40.39^a^	73.22 ± 9.67^a^	125.21 ± 21.26^a^^,^^c^	<0.001
LVESVI (mL/m^2^)	19.68 ± 3.35	60.60 ± 35.73^a^	24.13 ± 8.71^b^	89.06 ± 24.14^a^^,^^c^	<0.001
LVMI (g/m^2^)	33.89 ± 6.01	60.52 ± 19.79^a^	91.37 ± 30.21^a^^,^^b^	73.24 ± 17.48^a^	<0.001
Tissue characteristics					
T1ρ (ms)	44.67 ± 1.65	49.91 ± 3.65^a^	47.00 ± 1.70^a^	46.53 ± 1.64^a^^,^^b^	<0.001
Native T1 (ms)	1,283.35 ± 18.70	1,316.38 ± 52.68	1,321.98 ± 45.34	1,335.20 ± 50.33^a^	0.003
ECV (%)	-	33.42 ± 4.03	28.18 ± 2.23^b^	30.47 ± 2.54	<0.001

Data are expressed as mean ± standard deviation. Compared with the control group.

a *P* < 0.05; Compared with the ICM group.

b *P* < 0.05; Compared with the HCM group.

c *P* < 0.05. ICM, ischemic cardiomyopathy; HCM, hypertrophic cardiomyopathy; DCM, Dilated cardiomyopathy; LVEF, Left ventricular ejection fraction; LVEDVI, Left ventricular end-diastolic volume index; LVESVI, Left ventricular end-systolic volume index; LVMI, Left ventricular mass index; ECV, Extracellular volume.

**Figure 1:**
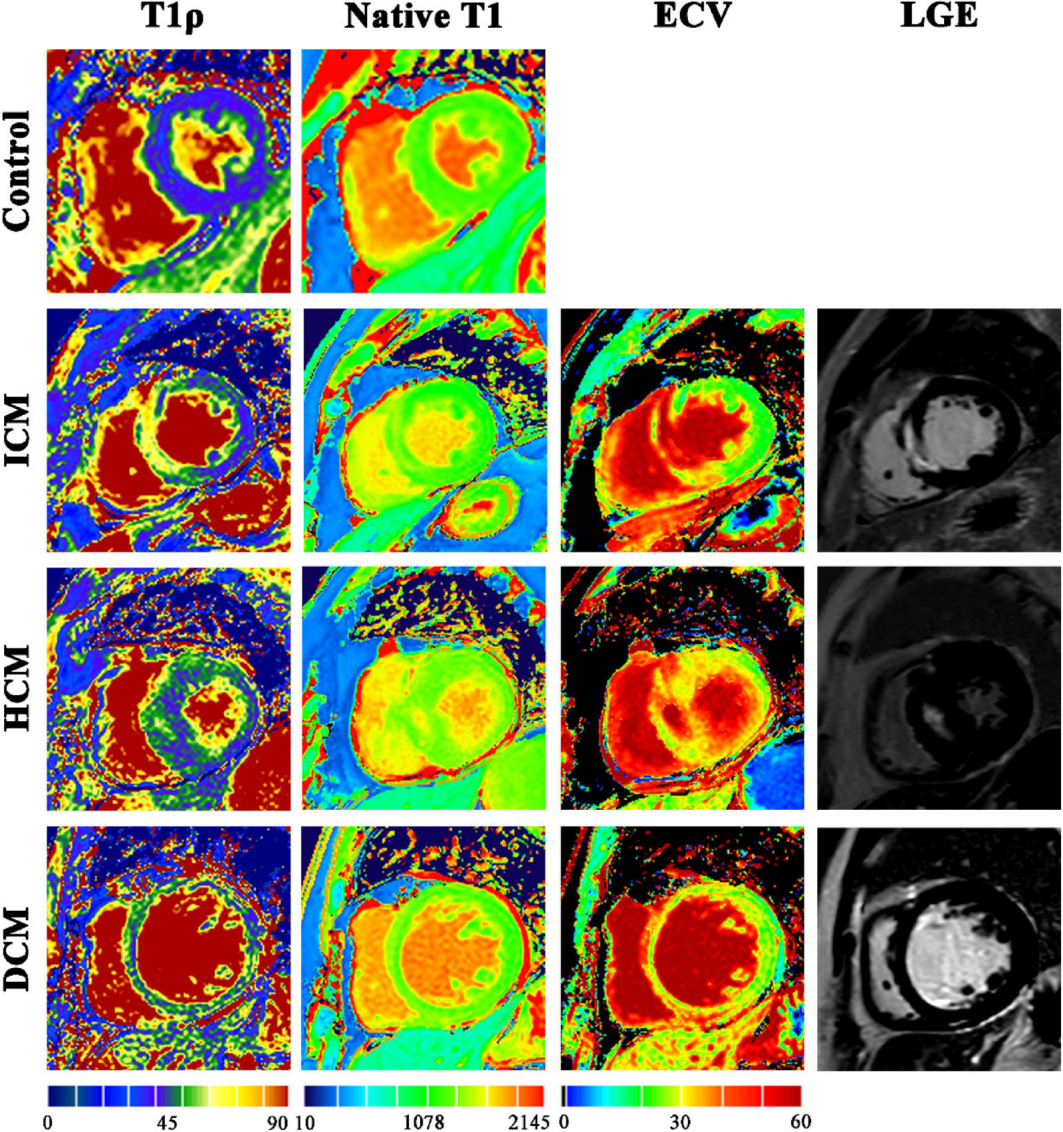
Example images of one healthy control and three patients. ICM, ischemic cardiomyopathy; HCM, hypertrophic cardiomyopathy; DCM, dilated cardiomyopathy; ECV, extracellular volume; LGE, late gadolinium enhancement.

In the entire study population, a total of 848 myocardial segments were acquired from T1ρ maps (624 and 224 segments in patients and controls), of which 68 segments excluded (8.02%) were mainly due to severe motion artifacts and inhomogeneity artifacts. The mean T1ρ value in our healthy controls (44.67 ± 1.65 ms) falls within the range of recently published normative data (approximately 43–45 ms) in healthy myocardium at 3 T using comparable spin-lock parameters (19). The T1ρ values were significantly higher in patients with ICM, HCM, and DCM compared with controls (all *P* < 0.05). ICM patients had higher T1ρ values than DCM patients. In addition, native T1 values were significantly higher in patients with DCM compared with controls (*P* < 0.05). The ICM group showed higher ECV values than the HCM group (*P* < 0.05) (Table 3). Representative examples of T1ρ maps alongside other CMR techniques from controls and patients with ICM, HCM, and DCM are shown in Figure 1.

At the myocardial segment level, significant differences in native T1 and T1ρ values were observed among the three types of segments (control, LGE– and LGE+) in all three groups (all *P* < 0.001). Specifically, T1ρ and native T1 values of segments with LGE were significantly higher in ICM, HCM and DCM patients compared with those in controls (all *P* < 0.001). The T1ρ and native T1 values of segments without LGE were significantly greater in ICM and DCM patients than those in controls (all *P* < 0.05), while in HCM patients, T1ρ values were higher but not significantly (*P* > 0.05). The T1ρ and native T1 values of segments with LGE were significantly higher in ICM patients compared with those without LGE (both *P* < 0.001), but no significant differences in DCM patients (both *P* > 0.05). In HCM patients, T1ρ values of segments with LGE were significantly higher compared with those without LGE (*P* < 0.001), but native T1 values were not (*P* > 0.05). Furthermore, the differences of ECV values between segments with and without LGE were significant in ICM, HCM and DCM patients (all *P* < 0.001) (Table 4).

**Table 4:** Comparison of tissue characteristics between healthy controls and patients based on segments.

Variable	Controls	LGE– segments	LGE + segments	*P* value
ICM				
N	206	138	85	
T1ρ (ms)	44.70 ± 3.48	47.08 ± 4.79^a^	54.47 ± 6.53^a^^,^^b^	<0.001
Native T1 (ms)	1,274.55 [1,253.36, 1,300.99]	1,298.53 [1,245.03, 1,344.98]^a^	1,332.90 [1,289.20, 1,396.40]^a^^,^^b^	<0.001
ECV (%)	-	29.77 ± 4.76	40.66 ± 9.33^b^	<0.001
HCM				
N	206	89	87	
T1ρ (ms)	44.70 ± 3.48	45.42 ± 2.96	48.21 ± 3.68^a^^,^^b^	<0.001
Native T1 (ms)	1,274.55 [1,253.36, 1,300.99]	1,307.16 [1,280.17, 1,347.08]^a^	1,314.77 [1,284.80, 1,370.63]^a^	<0.001
ECV (%)	-	26.88 ± 3.04	29.11 ± 3.88^b^	<0.001
DCM				
N	206	125	50	
T1ρ (ms)	44.70 ± 3.48	46.35 ± 4.01^a^	47.13 ± 3.70^a^	<0.001
Native T1 (ms)	1,274.55 [1,253.36, 1,300.99]	1,327.95 [1,293.87, 1,366.57]^a^	1,332.65 [1,302.18, 1,403.43]^a^	<0.001
ECV (%)	-	29.39 ± 3.02	33.92 ± 4.53^b^	<0.001

Data are expressed as mean ± standard deviation or median (25th to 75th percentile). Compared with segments in healthy controls.

a *P* < 0.05; Compared with segments without LGE.

b *P* < 0.05. ICM, ischemic cardiomyopathy; HCM, hypertrophic cardiomyopathy; DCM, dilated cardiomyopathy; LGE, late gadolinium enhancement; ECV, extracellular volume.

There was a significant positive correlation between ECV values and T1ρ values for ICM, HCM, and DCM patients (*r* = 0.598, 0.577, and 0.648, respectively; all *P* < 0.05), but no correlation between native T1 values and T1ρ values (all *P* > 0.05) (Table 5). At the myocardial segment level, ECV values were correlated positively with T1ρ values in all three groups (*r* = 0.481, 0.289, and 0.410, respectively; all *P* < 0.001). Additionally, segmental native T1 values were correlated with segmental T1ρ values for ICM (*r* = 0.343, *P* < 0.001) and DCM patients (*r* = 0.191, *P* = 0.011), but not for HCM patients (*P* = 0.264) (Table 5).

**Table 5:** Correlations between T1ρ and other tissue characteristics.

Variable	Global T1ρ (ms)		Segmental T1ρ (ms)	
	r	*P* value	r	*P* value
ICM				
Native T1 (ms)	0.370	0.175	0.343	<0.001
ECV (%)	0.598	0.018	0.481	<0.001
HCM				
Native T1 (ms)	0.276	0.385	0.085	0.264
ECV (%)	0.577	0.049	0.289	<0.001
DCM				
Native T1 (ms)	0.129	0.690	0.191	0.011
ECV (%)	0.648	0.023	0.410	<0.001

ICM, ischemic cardiomyopathy; HCM, hypertrophic cardiomyopathy; DCM, dilated cardiomyopathy; ECV, extracellular volume.

### ROC analysis

**Figure 2:**
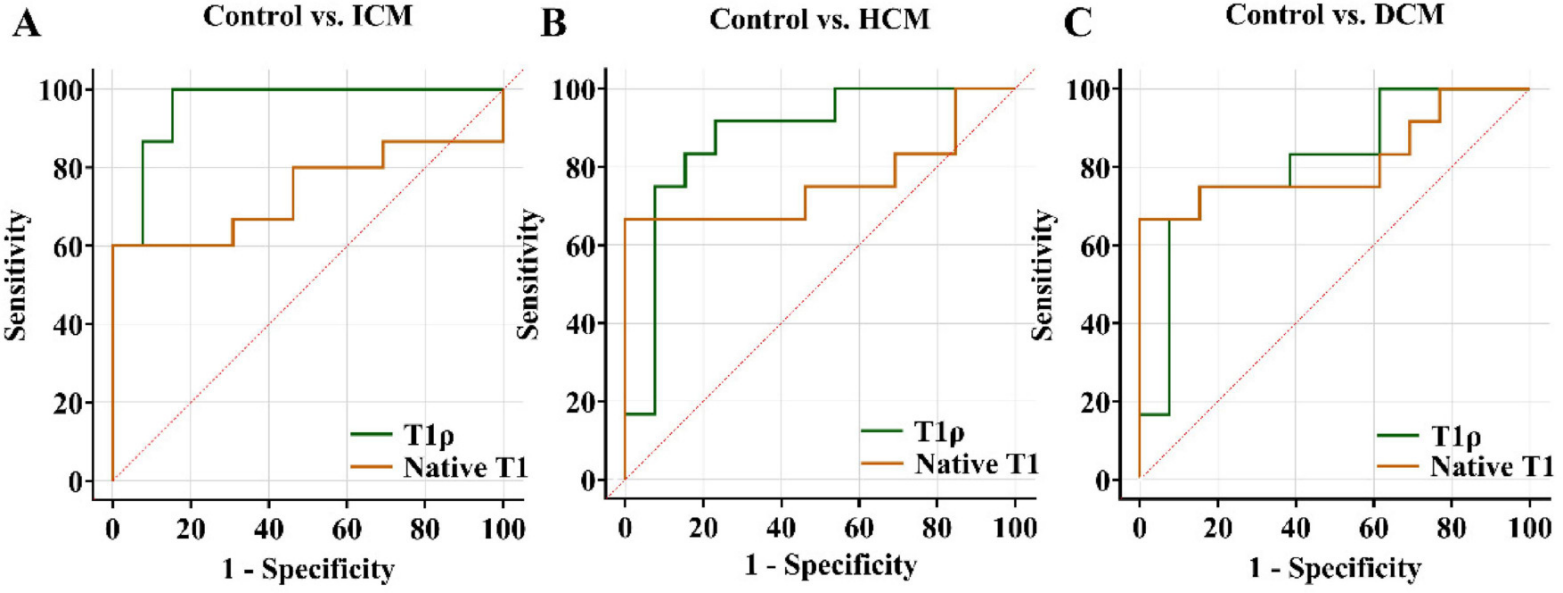
Receiver operating curves showing the diagnostic performance of T1ρ and native T1 in distinguishing between ICM patients and controls **(A)**, HCM patients and controls **(B)**, and DCM patients and controls **(C)** ICM, ischemic cardiomyopathy; HCM, hypertrophic cardiomyopathy; DCM, dilated cardiomyopathy.

On ROC analysis, T1ρ outperformed native T1 in differentiating both ICM patients [area under the curve (AUC): 0.959 vs. 0.739] and HCM patients (AUC: 0.878 vs. 0.763) from healthy controls. However, T1ρ and native T1 showed comparable performance for discriminating DCM patients from healthy controls (AUC: 0.814 vs. 0.814) (Figure 2).

**Figure 3:**
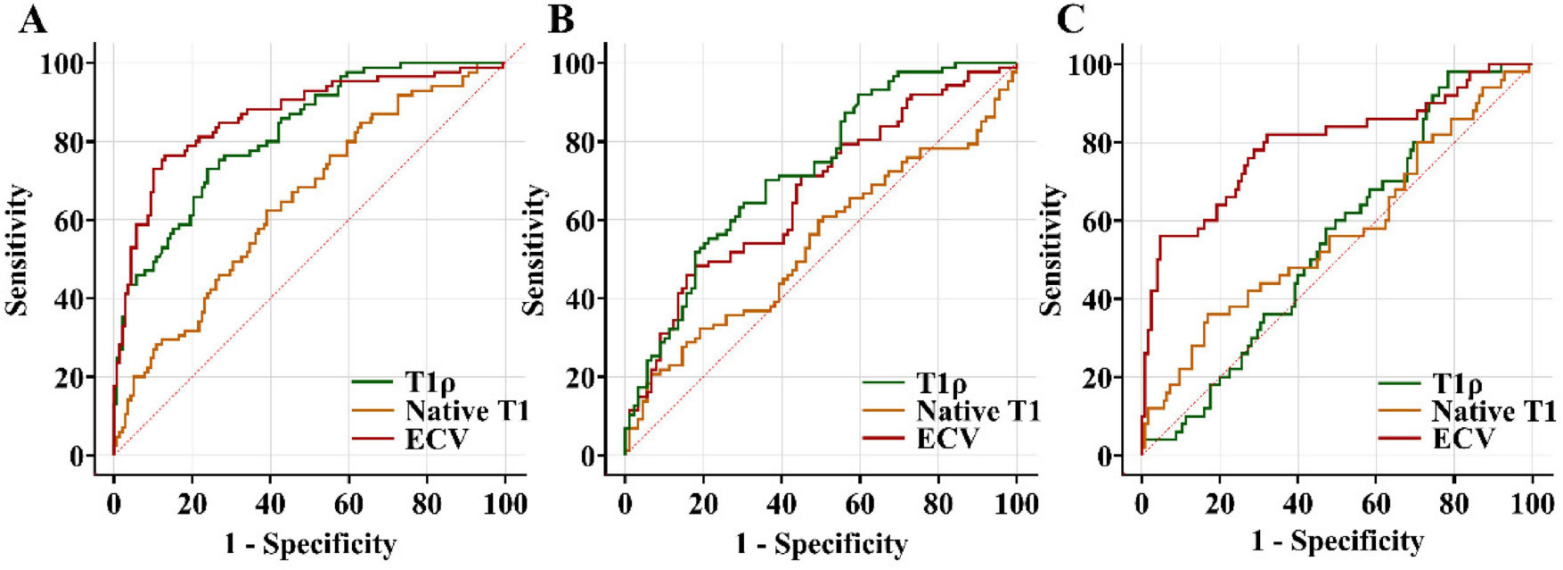
Receiver operating curves showing the diagnostic performance of T1ρ, native T1, and ECV in distinguishing LGE + segments from LGE− segments in patients with ischemic cardiomyopathy **(A)**, hypertrophic cardiomyopathy **(B)**, and dilated cardiomyopathy **(C)**. LGE, late gadolinium enhancement; ECV, extracellular volume.

Using GLMMs with random intercepts to account for the class effect due to multiple segments from the same individual or location, we assessed the associations between tissue characteristics and LGE. We found that T1ρ was significantly associated with the presence of LGE in ICM patients (*χ*^2^ = 72.748, *P* < 0.001) and demonstrated superior discriminative ability compared with native T1 (*χ*^2^ = 20.574, *P* < 0.001) in discriminating between LGE + and LGE− segments (AUC: 0.820 vs. 0.647, Table 6), but less discriminative than ECV (*χ*^2^ = 105.690, *P* < 0.001; AUC = 0.865). In HCM patients, T1ρ (*χ*^2^ = 18.378, *P* < 0.001) was the strongest discriminator (AUC = 0.726) compared with native T1 (*χ*^2^ = 3.229, *P* = 0.072; AUC = 0.539) and ECV (*χ*^2^ = 23.951, *P* < 0.001, AUC = 0.671; Table 6). For DCM patients, T1ρ (*χ*^2^ = 1.652, *P* = 0.199; AUC = 0.551) and native T1 (*χ*^2^ = 4.147, *P* = 0.042; AUC = 0.567) showed limited discriminative ability, while ECV (*χ*^2^ = 22.579, *P* < 0.001) demonstrated good discrimination (AUC = 0.795) (Table 6, Figure 3).

**Table 6:** Diagnostic performance of T1ρ and native T1 in differentiating LGE+ from LGE- segments.

Group	T1ρ AUC	Native T1 AUC	Difference (95% CI)	*P*-value
ICM	0.820	0.647	0.173 (0.095–0.251)	<0.001
HCM	0.726	0.539	0.187 (0.075–0.299)	0.001
DCM	0.551	0.567	−0.016(−0.129–0.097)	0.781

ICM, ischemic cardiomyopathy; HCM, hypertrophic cardiomyopathy; DCM, dilated cardiomyopathy; AUC, area under the curve; CI, confidence interval.

### Intra- and inter-observer variability

Table 7 summarizes the ICC values for T1ρ and native T1, demonstrating excellent intra-observer and inter-observer reproducibility.

**Table 7:** Intra- and inter-observer variability.

Variable	Intra-observer		Inter-observer	
	ICC	95% CI	ICC	95% CI
T1ρ (ms)	0.984	0.961–0.994	0.950	0.879–0.980
Native T1 (ms)	0.990	0.975–0.996	0.980	0.951–0.992

ICC, intraclass correlation coefficient; CI, confidence interval.

## Discussion

This exploratory study investigates the feasibility of T1ρ mapping for detecting myocardial fibrosis across distinct cardiomyopathy entities of ischemic and non-ischemic origin. Our findings demonstrate that T1ρ values are significantly higher in patients with ICM, HCM, and DCM compared with healthy controls. Compared with native T1, T1ρ showed favorable diagnostic performance in distinguishing ICM and HCM patients from healthy subjects, while both parameters exhibited comparable performance in DCM. Notably, elevated T1ρ values were observed in segments with or without LGE. T1ρ mapping demonstrates advantages in detecting local myocardial damage related to LGE imaging in ICM and HCM and exhibits a significant correlation with ECV across all disease types. These findings suggest that myocardial T1ρ mapping may serve as a non-contrast quantitative CMR approach for myocardial tissue characterization in structural heart diseases.

Native T1 mapping, which represents the intrinsic T1 relaxation time of myocardial tissue, served as a non-contrast CMR technique for characterizing and quantifying myocardial diffuse fibrosis (10). A recent meta-analysis has further demonstrated its prognostic value for predicting adverse outcomes in dilated cardiomyopathy (20). The T1ρ relaxation time reflects the decay of the longitudinal magnetization in the spin-lock radiofrequency field and is sensitive to low frequency interactions between water and macromolecules (8, 21). Several studies have reported the promise of T1ρ mapping in myocardial fibrosis detection, with myocardial T1ρ values in ischemic and non-ischemic cardiomyopathies being significantly higher than those in the controls (11, 13, 22–26). In our study, T1ρ values were significantly higher in patients with ICM, HCM and DCM compared with healthy control subjects. The native T1 values were significantly higher in patients with DCM compared with healthy controls consistent with other study (27). The T1ρ elevations observed in our patient cohorts (approximately 2–5 ms above controls) are within the range of reported differences in the literature. For HCM, Wang et al. reported T1ρ elevations of approximately 3–9 ms at 3 T depending on hypertrophy severity (23). For DCM, van Oorschot et al. reported elevations of approximately 4 ms (11). For native T1, our HCM and DCM values show elevations of approximately 39 ms and 52 ms above controls, respectively. These elevations are consistent with the magnitude reported by Dass et al., who demonstrated native T1 increases of 31–47 ms in these conditions (28). Absolute T1ρ values may vary across studies due to differences in field strength, spin-lock frequency, and sequence parameters, with the modest elevations reflecting the expected range for these patient populations. The mechanisms underlying elevated T1ρ remain incompletely understood, with potential contributions from factors such as chemical exchange, magnetization transfer, and increased water molecular motion and diffusion (21). Several factors beyond fibrosis may contribute to elevated T1ρ values, including myocardial edema (which increases free water content), technical factors such as field inhomogeneities and motion artifacts, and sequence-dependent variations in spin-lock parameters. These confounding factors should be considered when interpreting T1ρ elevations, particularly in the absence of histological validation. We found that T1ρ showed favorable diagnostic performance in distinguishing ICM or HCM patients from control subjects compared with native T1. In DCM patients, T1ρ and native T1 showed similar good performance. This discrepancy may be due to the different patterns of fibrosis in DCM, which may involve more diffuse and less focal fibrosis compared to ICM and HCM (29). The varying performance of T1ρ and T1 across different cardiomyopathies underscores the need for disease-specific validation and optimization of T1ρ mapping techniques.

LGE imaging allows visualization of myocardial replacement fibrosis, detection of which is important for the prognosis of patients with myocardial remodeling (3). To provide more information about myocardial damage associated with diverse disease progression, it is valuable to capture quantitative information from novel CMR techniques, such as T1ρ mapping, rather than relying solely on mere visualization. Previous studies have demonstrated the potential value of T1ρ mapping in detecting LGE as a noninvasive biomarker, based on the combined analysis of imaging and histological data (12, 30, 31). In our study, T1ρ values were significantly higher in segments with LGE compared with those in controls across all three disease types. Both native T1 and T1ρ values of LGE+ segments were significantly higher compared with LGE– segments for patients with ICM and HCM. The ROC analysis showed that, for ICM, T1ρ was more effective in distinguishing between LGE+ and LGE– segments compared with native T1. In HCM patients, T1ρ also outperformed native T1, though to a lesser extent than in ICM patients. However, LGE fails to detect diffuse interstitial fibrosis, which is common pattern in patients with DCM (29). In DCM patients, T1ρ and native T1 values were elevated in both LGE+ and LGE– segments, with no significant differences between them. Notably, elevated T1ρ values were observed across all three disease types even in the absence of detectable LGE, suggesting that T1ρ mapping may identify myocardial changes beyond focal fibrosis. Whether these changes represent diffuse fibrosis, edema, or other tissue alterations cannot be determined from the current data and requires further investigation with histological correlation. Clinical monitoring of these changes at the segmental level may eventually provide insights into disease progression, but larger longitudinal studies are needed to establish prognostic value. Further studies with a broader spectrum of cardiac conditions and different disease stages are required to validate these findings.

ECV is a well-recognized biomarker for myocardial diffuse fibrosis, with its correlation to fibrosis established through histological studies (10, 32). We found that there were significant positive correlations between the ECV and the T1ρ across all three disease types, suggesting that T1ρ values may provide similar information. Segmental measurements are able to capture local heterogeneity within the myocardium. The segmental ECV correlated positively with segmental T1ρ in all three patient groups. Despite the lack of correlation for global values, which was in keeping with previous findings (11, 24), there was a weak correlation between segmental native T1 and segmental T1ρ for DCM patients. The finding may be due to several factors, including the small patient sample with varying myocardial involvement, the influence of confounding variables on T1 values (9), and the potential for global measurements to obscure subtle regional differences. Further investigation into the mechanisms underlying T1ρ changes and their relationships with other well-established tissue characterizations in a larger sample size is needed to assess the value of T1ρ and facilitate its clinical implementation. While T1ρ demonstrated statistically significant differences between patient and control groups, and between LGE+ and LGE- segments, the clinical significance of these differences—particularly the modest elevations observed in some subgroups—remains to be established. Longitudinal studies with clinical endpoints are needed to determine whether T1ρ mapping provides incremental prognostic value beyond existing CMR biomarkers.

## Limitations

Several limitations should be acknowledged. First, the modest sample size, particularly within each disease subgroup, limits the generalizability of our findings. Second, there was no histological validation for the presence of myocardial fibrosis. In this study, LGE and ECV were used as reference biomarkers for myocardial fibrosis assessments, as they have been extensively validated against histology findings (32, 33). Third, potential confounding factors such as edema, technical artifacts, and sequence dependencies could not be fully controlled. Finally, the cross-sectional design prevents assessment of T1ρ's ability to track disease progression or treatment response, underscoring the need for future longitudinal studies with larger cohorts and histological correlation to validate our findings and establish the clinical utility of T1ρ mapping.

## Conclusion

In conclusion, this exploratory study demonstrates the potential of T1ρ mapping as a non-contrast CMR technique for detecting myocardial tissue alterations across ischemic and non-ischemic cardiomyopathies. T1ρ showed favorable diagnostic performance compared with native T1 in ICM and HCM, with comparable performance in DCM. These preliminary findings warrant validation in future studies with larger cohorts and histological correlation.

## Data Availability

The raw data supporting the conclusions of this article will be made available by the authors, without undue reservation.

## References

[1] Becker MAJ Cornel JH van de Ven PM van Rossum AC Allaart CP Germans T. The prognostic value of late gadolinium-enhanced cardiac magnetic resonance imaging in nonischemic dilated cardiomyopathy. JACC Cardiovasc Imaging. (2018) 11:1274–84. 10.1016/j.jcmg.2018.03.006 29680351

[2] Kwong RY Chan AK Brown KA Chan CW Reynolds HG Tsang S Impact of unrecognized myocardial scar detected by cardiac magnetic resonance imaging on event-free survival in patients presenting with signs or symptoms of coronary artery disease. Circulation. (2006) 113:2733–43. 10.1161/CIRCULATIONAHA.105.570648 16754804

[3] Kim R Wu E Rafael A Chen EL Parker MA Simonetti O The use of contrast-enhanced magnetic resonance imaging to identify reversible myocardial dysfunction. N Engl J Med. (2000) 343:1445–53. 10.1056/NEJM200011163432003 11078769

[4] Hunold P Schlosser T Vogt FM Eggebrecht H Schmermund A Bruder O Myocardial late enhancement in contrast-enhanced cardiac MRI: distinction between infarction scar and non-infarction-related disease. Am J Roentgenol. (2005) 184:1420–6. 10.2214/ajr.184.5.01841420 15855089

[5] Fraum TJ Ludwig DR Bashir MR Fowler KJ. Gadolinium-based contrast agents: a comprehensive risk assessment. J Magn Reson Imaging. (2017) 46:338–53. 10.1002/jmri.25625 28083913

[6] Gulani V Calamante F Shellock FG Kanal E Reeder SB. Gadolinium deposition in the brain: summary of evidence and recommendations. Lancet Neurol. (2017) 16:564–70. 10.1016/S1474-4422(17)30158-8 28653648

[7] Messroghli DR Moon JC Ferreira VM Grosse-Wortmann L He T Kellman P Clinical recommendations for cardiovascular magnetic resonance mapping of T1, T2, T2* and extracellular volume: a consensus statement by the society for cardiovascular magnetic resonance (SCMR) endorsed by the European association for cardiovascular imaging (EACVI). J Cardiovasc Magn Reson. (2016) 19:75. 10.1186/s12968-017-0389-8 PMC5633041 28992817

[8] Bustin A Witschey WRT van Heeswijk RB Cochet H Stuber M. Magnetic resonance myocardial T1ρ mapping. J Cardiovasc Magn Reson. (2023) 25:34. 10.1186/s12968-023-00940-1 37331930 PMC10278347

[9] Puntmann VO Peker E Chandrashekhar Y Nagel E. T1 mapping in characterizing myocardial disease. Circ Res. (2016) 119:277–99. 10.1161/CIRCRESAHA.116.307974 27390332

[10] Haaf P Garg P Messroghli DR Broadbent DA Greenwood JP Plein S. Cardiac T1 mapping and extracellular volume (ECV) in clinical practice: a comprehensive review. J Cardiovasc Magn Reson. (2016) 18:89. 10.1186/s12968-016-0308-4 27899132 PMC5129251

[11] van Oorschot JWM Güçlü F de Jong S Chamuleau SAJ Luijten PR Leiner T Endogenous assessment of diffuse myocardial fibrosis in patients with T1ρ-mapping. J Magn Reson Imaging. (2017) 45:132–8. 10.1002/jmri.25340 27309545

[12] Witschey WRT Zsido GA Koomalsingh K Kondo N Minakawa M Shuto T *In vivo* chronic myocardial infarction characterization by spin locked cardiovascular magnetic resonance. J Cardiovasc Magn Reson. (2012) 14:48. 10.1186/1532-429X-14-37 22704222 PMC3461454

[13] Thompson EW Kamesh Iyer S Solomon MP Li Z Zhang Q Piechnik S Endogenous T1ρ cardiovascular magnetic resonance in hypertrophic cardiomyopathy. J Cardiovasc Magn Reson. (2021) 23:120. 10.1186/s12968-021-00813-5 34689798 PMC8543937

[14] Elliott PM Anastasakis A Borger MA Borggrefe M Cecchi F Charron P 2014 ESC guidelines on diagnosis and management of hypertrophic cardiomyopathy. Eur Heart J. (2014) 35:2733–79. 10.1093/eurheartj/ehu284 25173338

[15] Zhuang B Li S Xu J Zhou D Yin G Zhao S Age- and sex-specific reference values for atrial and ventricular structures in the validated normal Chinese population: a comprehensive measurement by cardiac MRI. J Magn Reson Imaging. (2020) 52:1031–43. 10.1002/jmri.27160 32243664

[16] Pinto YM Elliott PM Arbustini E Adler Y Anastasakis A Böhm M Proposal for a revised definition of dilated cardiomyopathy, hypokinetic non-dilated cardiomyopathy, and its implications for clinical practice: a position statement of the ESC working group on myocardial and pericardial diseases. Eur Heart J. (2016) 37:1850–8. 10.1093/eurheartj/ehv727 26792875

[17] Witschey WRT Borthakur A Elliott MA Mellon E Niyogi S Wallman DJ Artifacts in T1ρ-weighted imaging: compensation for B1 and B0 field imperfections. J Magn Reson. (2007) 186:75–85. 10.1016/j.jmr.2007.01.015 17291799 PMC1995435

[18] Mosteller RD. Simplified calculation of body-surface area. N Engl J Med. (1987) 317:1098. 10.1056/NEJM198710223171717 3657876

[19] Han C Xu H Gao H Liu F Wu J Liu Y Effect of spin-lock frequency on quantitative myocardial T1rho mapping. Insights Imaging. (2024) 15:176. 10.1186/s13244-024-01762-0 38992330 PMC11239636

[20] Marchini F Dal Passo B Campo G Tonet E Serenelli M Cossu A T1 mapping and major cardiovascular events in non-ischaemic dilated cardiomyopathy: a systematic review and meta-analysis. ESC Heart Fail. (2025) 12:2621–30. 10.1002/ehf2.15279 40285366 PMC12287780

[21] Han Y Liimatainen T Gorman RC Witschey WRT. Assessing myocardial disease using T1ρ MRI. Curr Cardiovasc Imaging Rep. (2014) 7:9248. 10.1007/s12410-013-9248-7 24688628 PMC3968806

[22] Wang K Zhang Y Zhang W Jin H An J Cheng J Role of endogenous T1ρ and its dispersion imaging in differential diagnosis of cardiac amyloidosis. J Cardiovasc Magn Reson. (2024) 26:101080. 10.1016/j.jocmr.2024.101080 39127261 PMC11422604

[23] Wang K Zhang W Li S Jin H Jin Y Wang L Noncontrast T1ρ dispersion imaging is sensitive to diffuse fibrosis: a cardiovascular magnetic resonance study at 3 T in hypertrophic cardiomyopathy. Magn Reson Imaging. (2022) 91:1–8. 10.1016/j.mri.2022.05.001 35525524

[24] Bustin A Pineau X Sridi S van Heeswijk RB Jaïs P Stuber M Assessment of myocardial injuries in ischaemic and non-ischaemic cardiomyopathies using magnetic resonance T1-rho mapping. Eur Heart J Cardiovasc Imaging. (2024) 25:548–57. 10.1093/ehjci/jead319 37987558 PMC10966324

[25] Bustin A Toupin S Sridi S Yerly J Bernus O Labrousse L Endogenous assessment of myocardial injury with single-shot model-based non-rigid motion-corrected T1 rho mapping. J Cardiovasc Magn Reson. (2021) 23:119. 10.1186/s12968-021-00781-w 34670572 PMC8529795

[26] Dong Z Yin G Yang K Jiang K Wu Z Chen X Endogenous assessment of late gadolinium enhancement grey zone in patients with non-ischaemic cardiomyopathy with T1ρ and native T1 mapping. Eur Heart J Cardiovasc Imaging. (2023) 24:492–502. 10.1093/ehjci/jeac128 35793269

[27] Puntmann VO Voigt T Chen Z Mayr M Karim R Rhode K Native T1 mapping in differentiation of normal myocardium from diffuse disease in hypertrophic and dilated cardiomyopathy. JACC Cardiovasc Imaging. (2013) 6:475–84. 10.1016/j.jcmg.2012.08.019 23498674

[28] Dass S Suttie JJ Piechnik SK Ferreira VM Holloway CJ Banerjee R Myocardial tissue characterization using magnetic resonance noncontrast t1 mapping in hypertrophic and dilated cardiomyopathy. Circ Cardiovasc Imaging. (2012) 5:726–33. 10.1161/CIRCIMAGING.112.976738 23071146

[29] Eijgenraam TR Silljé HHW de Boer RA. Current understanding of fibrosis in genetic cardiomyopathies. Trends Cardiovasc Med. (2020) 30:353–61. 10.1016/j.tcm.2019.09.003 31585768

[30] van Oorschot JWM El Aidi H Jansen of Lorkeers SJ Gho JMIH Froeling M Visser F Endogenous assessment of chronic myocardial infarction with T1ρ-mapping in patients. J Cardiovasc Magn Reson. (2015) 16:104. 10.1186/s12968-014-0104-y PMC4272542 25526973

[31] Stoffers RH Madden M Shahid M Contijoch F Solomon J Pilla JJ Assessment of myocardial injury after reperfused infarction by T1ρ cardiovascular magnetic resonance. J Cardiovasc Magn Reson. (2016) 19:17. 10.1186/s12968-017-0332-z PMC5310026 28196494

[32] Miller CA Naish JH Bishop P Coutts G Clark D Zhao S Comprehensive validation of cardiovascular magnetic resonance techniques for the assessment of myocardial extracellular volume. Circ Cardiovasc Imaging. (2013) 6:373–83. 10.1161/CIRCIMAGING.112.000192 23553570

[33] Iles LM Ellims AH Llewellyn H Hare JL Kaye DM McLean CA Histological validation of cardiac magnetic resonance analysis of regional and diffuse interstitial myocardial fibrosis. Eur Heart J Cardiovasc Imaging. (2014) 16:14–22. 10.1093/ehjci/jeu182 25354866

